# Oxidative Stress in Dairy Cows: Insights into the Mechanistic Mode of Actions and Mitigating Strategies

**DOI:** 10.3390/antiox10121918

**Published:** 2021-11-29

**Authors:** Aurele Gnetegha Ayemele, Mekonnen Tilahun, Sun Lingling, Samy Abdelaziz Elsaadawy, Zitai Guo, Gaojuan Zhao, Jianchu Xu, Dengpan Bu

**Affiliations:** 1 State Key Laboratory of Animal Nutrition, Institute of Animal Sciences, Chinese Academy of Agricultural Sciences, Beijing 100193, China; ayemeleaurel@yahoo.com (A.G.A.); dmtilahun84@gmail.com (M.T.); sunlingling114289@163.com (S.L.); samyelsaadawy@outlook.com (S.A.E.); i.am@guozitai.com (Z.G.); 2 Honghe Center for Mountain Futures, Kunming Institute of Botany, Chinese Academy of Sciences, Honghe County 654400, China; zhaogaojuan@mail.kib.ac.cn; 3 World Agroforestry Center, East and Central Asia, Kunming 650201, China; 4 Joint Laboratory on Integrated Crop-Tree-Livestock Systems of the Chinese Academy of Agricultural Sciences (CAAS), Ethiopian Institute of Agricultural Research (EIAR) and World Agroforestry Center (ICRAF), Beijing 100193, China

**Keywords:** antioxidants genes, feed additives, immune function, pathogenic microbial cells, pro-inflammatory genes

## Abstract

This review examines several molecular mechanisms underpinning oxidative stress in ruminants and their effects on blood and milk oxidative traits. We also investigate strategies to alleviate or repair oxidative damages by improving animal immune functions using novel feed additives. Microbial pathogenic cells, feeding management, and body condition score were some of the studied factors, inducing oxidative stress in ruminants. The predominance of *Streptococcus* spp. (24.22%), *Acinetobacter* spp. (21.37%), *Romboutsia* spp. (4.99%), *Turicibacter* spp., (2.64%), *Stenotrophomonas* spp. (2.33%), and *Enterococcus* spp. (1.86%) was found in the microbiome of mastitis cows with a decrease of d-mannose and increase of xanthine:guanine ratio when *Streptococcus* increased. Diversity of energy sources favoring the growth of *Fusobacterium* make it a keystone taxon contributing to metritis. Ruminal volatile fatty acids rose with high-concentrate diets that decreased the ruminal pH, causing a lysis of rumen microbes and release of endotoxins. Moreover, lipopolysaccharide (LPS) concentration, malondialdehyde (MDA), and superoxide dismutase (SOD) activities increased in high concentrate cows accompanied by a reduction of total antioxidant capacity (T-AOC), glutathione peroxidase (GPx), and catalase (CAT) activity. In addition, albumin and paraoxonase concentrations were inversely related to oxidative stress and contributed to the protection of low-density and high-density lipoproteins against lipid peroxidation, protein carbonyl, and lactoperoxidase. High concentrate diets increased the expression of MAPK pro-inflammatory genes and decreased the expression of antioxidant genes and proteins in mammary epithelial tissues. The expression levels of NrF2, NQO1, MT1E, UGT1A1, MGST3, and MT1A were downregulated, whereas NF-kB was upregulated with a high-grain or high concentrate diet. Amino-acids, vitamins, trace elements, and plant extracts have shown promising results through enhancing immune functions and repairing damaged cells exposed to oxidative stress. Further studies comparing the long-term effect of synthetic feed additives and natural plant additives on animal health and physiology remain to be investigated.

## 1. Introduction

Molecular oxygen is an important electron acceptor of the biosphere, featuring a bi-radical structure capable of gaining unpaired electrons, leading to reduced species commonly known as reduced or reactive oxygen species (ROS), such as superoxide (O_2_^•−^), hydrogen peroxide (H_2_O_2_), hydroxyl (HO^•^), peroxyl (ROO^•^), and alkoxyl (RO^•^) radicals [1]. With the presence of oxygen and water in the biosphere, humans and animals are exposed to two main sources of ROS. First, ionizing cosmic or ground radiation and pollution smoke modify the chemical structure of oxygen and water, creating free radicals, including the highly reactive hydroxyl group [2,3]. Second, after the death products of pathogens, macrophage activities also conduct ROS throughout the electron transport chain, forming complex I and II that release the pro-inflammatory transcription factors NFκB and AP-1 [4]. Those transcription factors contribute to upregulate the expression of pro-inflammatory chemokines or cytokines and adhesion molecules [4].

A lot of diseases, especially degenerative ones, are now considered at least partly caused by oxidative damages. The nucleic acids, proteins, and lipids are the cell’s organic components that are chemically damaged by ROS [5,6]. First, the most reactive hydroxyl group will interact with both the bases and sugars (ribose and desoxyribose) to change the nucleotide structure and functions, leading to mutation and degenerative disorders. At the same time, the change in sugar structure will also break down DNA strands. Second, when the SH-HS structure of thiols protein, considered the major protein antioxidant, is oxidized into disulfite S-S, irreversible protein damaging consists of SOH, SO_2_H, and SO_3_H synthesis, impairing cellular calcium homeostasis [7]. Third, the juxtaposition of the poly-unsaturated fatty acids on the cell membrane propagates their oxidation in the presence of ROS, thereby destroying entire cellular membranes via chain reaction [8]. Subsequently, the membrane function will be disturbed and the integrity of the physical structure of the cellular compartmentalization cannot be maintained, leading to enzymes leakage, electrolyte diffusion, metals, and small molecules transiting [9,10]. The diffused products of lipid peroxidation, such as 4-hydroxynonenal, are also responsible for protein and nucleic acids alterations, including their related functions.

A study using redox Western analysis and redox-sensitive green fluorescent proteins to investigate the redox signaling and oxidative stress in different subcellular compartments revealed that the redox status from most reducing to most oxidizing was as follows: mitochondria > nuclei > cytoplasm > endoplasmic reticulum > extracellular space [11]. The relatively alkaline pH (near 8.0) of mitochondria makes its protein thiols vulnerable to apoptosis and necrosis. Furthermore, the relatively reduced nuclear redox state is a key transcriptional factor in response to oxidative stress. Animal metabolic and physiological traits respond quite differently to different oxidative stress factors and management strategies, but the specific cellular mechanisms of action are not often described. Some oxidative stress factors considered and described within this study are the microbial pathogenic cells inducing mastitis and metritis degenerative diseases in dairy cows along with feeding management using different concentrates: forage ratios, with effects on some expressed genes associated with oxidative stress pathways. To address these drawbacks, several studies have been carried out on the dietary supplementation with additives like methionine and lysine amino-acids to evaluate the effects on the cattle oxidative status [12,13,14]. Meanwhile, very few feed additives have been tested as immune function bioregulators, and the combination effect of methionine and lysine as well as vitamins, trace elements, and plant extracts are not commonly studied. This study reviews the cellular mechanisms that induce oxidative stress under different conditions as well as the effects on milk and blood oxidative traits in ruminants. The recovering molecular pathways after supplementing different feed additives such as methionine-lysine combination, l-arginine, *N*-carbamylglutamate, plant extracts, vitamins, and trace elements as alleviating strategies are described.

## 2. Microbial Activities as Driver of Degenerating Oxidative Stress Diseases and Mechanisms of Action

### 2.1. Mastitis

Mastitis is an intra-mammary infection driven by host-pathogen interactions that causes severe economic losses, including decrease in milk production and quality, premature culling, lower conception rates, and treatment costs in dairy cattle [15,16]. Therefore, host-microbial interactions have been studied, aiming to optimize the lactating performance of cows [17] and depending on the pathogenic microbes primary reservoir and mode of transmission, mastitis has been categorized into contagious and environmental forms. Some detrimental microbes such as *Streptococcus dysgalactiae*, *Streptococcus uberis*, *Escherichia coli*, and *Klebsiella*, non-aureus *Staphylococci* (NAS), *Staphylococcus aureus*, and *Enterococcus* spp. have been correlated with mastitis occurrence [18], but unfortunately, the mechanisms underpinning host–microbial interactions inducing mastitis are unclear. The invasion stage mainly occurs from environmental microbes through the mammary teat canal opening in the direction of the tissues, then to the epithelium cells of the duct, causing inflammation and development of granulation tissues that finally appear as polypoid swelling [19]. Pathogens in the epithelium that remain undestroyed by immune cells or neutrophils will cause oedema, leading to a vacuolated and desquamated epithelial acini [20]. After invasion, the pathogenic bacteria undergo a rapid multiplication, leading to reductions in healthy milk secretory tissue, scanty milk with blood traces as well as gangrene and thrombosis damaging the udder tissue, which can potentially lead to toxaemia and death in acute cases [21]. Mastitis milk is source of zoonosis like tuberculosis, brucellosis, and gastroenteritis. A number of previous studies analyzed the microbiome of mastitis cows compared to healthy ones, indicating a lower microbial diversity in mastitis bovine milk [22,23], quantified by a lower Shannon index. Indeed, at the phylum level, fewer *Proteobacteria*, *Actinobacteria,* and *Acidobacteria* were found, with a predominance of the following genera: *Streptococcus* spp. (24.22%), *Acinetobacter* spp. (21.37%), *Romboutsia* spp. (4.99%), *Turicibacter* spp. (2.64%), *Stenotrophomonas* spp. (2.33%), *Enterococcus* spp. (1.86%), *Microbacterium* spp. (1.66%), *Aerococcus* spp. (1.60%), *Corynebacterium-1* spp. (1.49%), and *Bacteroides* spp. (1.44%) [24]. Tong et al. (2019) [24] describes the correlation analysis between milk metabolites of the intra-mammary infected cows and their microbial populations. Indeed, d-mannose tended to decrease when the pathogenic *Streptococcus* increased. This situation definitely impairs the potency of d-mannose known to regulate these microbial infections. Moreover, xanthine with its catalytic xanthine oxidase enzyme can first produce uric acid, then reactive oxygen species, superoxide radicals, and hydrogen peroxide, all of which are involved in pathogenesis. This explains how *Streptococcus*, positively correlated to the xanthine:guanine ratio, can gradually induce severe oxidative stress in dairy cattle. Meanwhile, other bacteria from *Lachnospiraceae*, *Lactobacillaceae*, *Xanthomonadaceae*, *Microbacteriaceae*, and *Brevibacteriaceae* families were negatively correlated with the xanthine:guanine ratio. Quinic acid, with its beneficial effects, was negatively correlated to detrimental *Enteroccocaceae*. These outcomes could provide some guidelines on manipulating microbiota to reduce incidents of oxidative stress.

Generally, mastitis cases are recorded when the somatic cell counts (SCC) exceed the tolerated value [25]. Over time, if the invading mastitis-pathogens are killed, then the somatic cells return to their normal range. Therefore, genetic selection for reducing excess SCC occurrence may improve resistance to mastitis, no matter the causative pathogen species. Meanwhile, different pathogens contribute to different infection extents. A genetic correlation analysis revealed that *E. coli* had a lower incidence on SCC inducing mastitis when compared with *Strep. dysgalactiae*, NAS, and *Strep. Uberis* [18]. Cows with these pathogens showed higher lactation SCC, infecting them for a longer period than cows infected by *E. coli* [26], which is purely of environmental origin [27], and that also makes it less severe for the herd compared to the other pathogens that are contagious within the herd [28]. Moreover, *Staphylococcus aureus* is known to induce chronic mammary infections [29], because it avoids being killed by macrophages, reducing the potency of the mammary immune system [30], and leading to the production of virulence factors [31]. The use of lactation SCC as an indicator trait for biomonitoring of cows exposed to mammary inflammation shows promise in mitigating the inflammation of mammary tissues through technological manipulation of involved microbes.

### 2.2. Metritis

Uterine wall inflammation known as metritis or pelvic inflammatory disease mostly occurs in negative energy balance (NEB) conditions. Closely examining the microbiological determinants of this physiological disorder, the increase in *Fusobacterium*, *Bacteroides*, and *Porphyromonas* has been associated with metritis [32,33], while a reduction in the same bacteria has been associated with the cure of metritis regardless of the antibiotic treatment [34]. Therapy with ceftiofur against metritis led to a reduction in *Fusobacterium*, suggesting that *Fusobacterium* and especially *F. necrophorum* is a keystone pathogen that can stabilize dysbiotic metritis microbiota [35]. Therefore, special attention should be paid to this taxa when planning mitigation strategies. Out of 95 carbon energy sources investigated that may promote the growth of *Fusobacterium*, up to 47 were metabolized by *F. necrophorum*, enabling it to proliferate in postpartum cows using an array of energy sources, even in animal NEB [36]. For instance, in iron-limited conditions where *F. necrophorum* thrived, an increase of the expression of virulence genes such as the glycoprotein of influenza virus called haemagglutinin and leukotoxin genes was reported (2.49 and 3.72 fold increase respectively [37]). Meanwhile, *F. necrophorum* encoded Haemolysin, yebN homologue, and *tonB* homologue do not cause significant lysis of the red blood cell, but greatly contribute to nutrients acquisition, and these were pronouncedly down-regulated [37]. β-Hydroxybutyric acid (BHBA) is recognized as a preferred source of energy of *Fusobacterium*, particularly after calving, when cows undergo NEB and feed intake alone cannot meet their energy demands. This deficit results in lipid mobilization from adipose tissue in the form of non-esterified fatty acids (NEFA), uptake of NEFA, and partial oxidation of NEFA in the liver, which forms large quantities of BHBA that end up in the blood stream [38]. Metritis cows generally have higher NEFA and BHBA concentrations than healthy cows, and the higher NEFA and BHBA levels impaired leukocyte functions [39]. In the uterus, l-glutamine and pyruvic acid metabolites contribute little to the growth of *F. necrophorum* during early lactation, but they are importantly used in mammary glands for the uptake for milk production [40]. Uterine energy metabolites could therefore serve as biomarkers of host immunity and uterine pathogen growth. Coenzyme A (CoA) and its thioester derivatives are synthesized by prokaryotes and eukaryotes cells and are involved in major metabolic pathways, including the regulation of gene expression and redox activity known as protein CoAlation, strongly induced in response to oxidizing agents and metabolic stresses in exponentially growing bacteria to prevent overoxidation [41].

## 3. Molecular Mechanism of Oxidative Stress and Feeding Management Factors Associated with Body Condition Score (BCS)

Nutritional practices can provide antioxidants acting to lower pro-oxidant loads and decrease the incidence of oxidative stress. Therefore, nutritional oxidative stress occurs when there is an insufficient supply of nutrients and bioactive compounds to provide defenses against the degenerative oxidants activities through the increase of free radicals [42,43]. Apoptosis is a form of programmed cell death that is initiated and completed in a systematic manner by triggering and/or synthesizing the necessary gene products for cell death [44]. Berlier et al. (2015) [45] established that members of the MAPK (mitogen-activated protein kinase) family, such as c-Jun *N*-terminal kinase (JNK), extracellular signal-regulated kinase 1/2 (ERK1/2), and p38 MAPK all play critical roles in a signaling pathways mediating oxidative stress-induced apoptosis. Although ROS serve a variety of physiological functions [46], oxidative stress occurs when excessive production is not countered by antioxidant mechanisms, resulting in potentially pathological changes [47]. In low concentrations, ROS contribute to several physiological functions but they can be detrimental for living cells if they are present in excessive amounts that cannot be overridden by available antioxidants [48]. Excess ROS can deplete antioxidant enzyme activity and overwhelm the cell’s intrinsic antioxidant defense mechanisms, resulting in oxidative stress, lipid peroxidation, and apoptosis [49,50]. Abuelo et al. (2015) [51] also proposed that nutrition plays a crucial role in free radical-mediated lipid peroxidation, which is vital in high-yielding cows that are naturally susceptible to oxidative stress. Blood metabolites are well known to reflect an animal’s nutritional status as well as its physiological status [52]. To maintain a low level of ROS production, tissues are equipped with a robust antioxidant defense system that includes endogenous non-enzymatic antioxidants (e.g., albumin, bilirubin, and glutathione), enzymatic antioxidants (e.g., superoxide dismutase: SOD, catalase: CAT, and glutathione peroxidase: GPx), and exogenous antioxidants (e.g., tocopherols and carotenoids) [53]. Different dietary backgrounds as well as different body condition score ranges affect oxidative stress status differently, with specific implications on biological traits of milk and blood, mediated by specific molecular pathways.

### 3.1. High Concentrate Feed Effect on Oxidative Stress and Molecular Pathways

Dairy cows need an adequate intake of coarse fiber to ensure proper rumination, saliva processing, and rumen buffering [54]. As a result, it is recommended that at least 40% of the feed particles containing dietary ingredients in total mixed rations (TMR) for dairy cows be larger than 8 mm [55]. However, commercial cow lactation diets are typically high in concentrate to maximize nutrient intake and milk production efficiency, resulting in diets with low or moderate physically effective neutral detergent fiber (PeNDF) [56]. However, high-concentrate diets are likely to result in metabolic and systemic dysfunction [57], leading to a rise in the concentration of ruminal volatile fatty acids and a corresponding decrease in ruminal pH [58]. Therefore, subacute ruminal acidosis (SARA) will occur, especially when the rumen pH stays depressed for extended periods of time per day, e.g., 5.6 for > 3 h/day [59,60]. SARA frequency on dairy farms in Wisconsin has been reported to range between 19 and 26% in early to mid-lactation cows [61]. Additionally, Kleen et al. (2013) [62] observed a 20% frequency in 315 cows in Northern Germany. Furthermore, it is thought that low pH causes a lysis of rumen microbes, release of endotoxins lipopolysaccharide (LPS) from Gram-negative bacteria, and increases the permeability of the rumen barrier [63]. LPS in the rumen fluid is absorbed mostly by the rumen wall, then travels through the ruminal veins to the liver via the portal vein [64,65], and subsequently reaches the mammary gland, triggering inflammatory responses that result in decreased production [66]. In some tested dairy cattle fed with high concentrate, the oxidant and anti-oxidant biomarkers such as LPS concentration in the rumen fluid, hepatic vein plasma, portal vein plasma, and jugular vein plasma were higher than in cattle fed low concentrate (Table 1). In addition, malondialdehyde (MDA) known as a biomarker for detrimental lipid peroxidation and SOD activities, increased in high concentrate cows accompanied with a reducing of total antioxidant capacity (T-AOC), GPx, and CAT activity. Regarding the composition and structure of milk metabolites, blood metabolites, hormones, and enzymes, a substantial difference was observed in high concentrate compared to low concentrate cattle. Aviram and Rosenblat (2004) [67] reported that albumin and paraoxonase concentrations are inversely related to oxidative stress, partly because they contribute to the protection of low-density lipoprotein and high-density lipoprotein against lipid peroxidation, along with protein carbonyl and lactoperoxidase. Increased LPS levels in portal and hepatic veins further damage hepatocytes and impair liver function, shown by enhanced TNF receptor-associated factor 6 (TRAF6), p-NF-B, p38 MAPK, IL-1, and serum amyloid A (SAA) levels in the liver [68]. Moreover, LPS increases the concentration of LPS-binding protein (LBP), serum amyloid A (SAA), and haptoglobin (HP) in peripheral blood during SARA [69]. Furthermore, high concentrations of LPS can induce the production of ROS by Kupffer cells and neutrophils. Inadequate regulation of ROS accumulation within metabolically active tissues results in oxidative stress [70]. Isoprostanes (IsoP) are important biomarkers of lipid peroxidation damage, because they form when ROS oxidize arachidonic acid. Indeed, IsoP was found in the blood and milk of dairy cows during periods of high oxidative stress, such as the peripartum and mastitis periods [71].

Molecular mechanisms triggered after feeding high-concentrate inducing SARA in dairy cattle exhibited changes in oxidative stress parameters, including the genomic signaling pathways in different compartments (mammary gland and epithelial tissue, liver, hind-gut, and uterus) (Table 2). Indeed, high concentrate diets increased the expression of MAPK pro-inflammatory genes and decreased the expression of antioxidant genes and proteins in mammary epithelial tissue, especially the nuclear factor E2-related factor 2 (NrF2). The relative mRNA abundances of GPx1 and 3, CAT, and SOD2 were significantly lower in liver and epithelial tissues of cows fed a high-grain diet than those fed a low-grain diet. The expression levels of NrF2, NQO1, MT1E, UGT1A1, MGST3, and MT1A were downregulated, whereas NF-kB was upregulated with a high-grain or concentrate diet. Moreover, NrF2 total protein and mRNA levels decreased in low-grains diet. From the tested animals, the high concentrate diet inducing LPS led to the suppression of cellular antioxidant defense capacity, which triggered increased oxidative stress, suggesting that the Nrf2-dependent antioxidant response might be affected by higher levels of LPS translocated to the bloodstream.

### 3.2. Body Condition Score Status Effect on Oxidative Stress

The body condition score (BCS) is a simple, practical, and accurate tool for assessing energy and fat reserves of dairy cows [81,82] and identifying animals at increased risk of developing postpartum disorders [82,83,84]. Milk yield, herd health, reproductive performance, and overall farm profitability can all be improved by managing the BCS of dairy cows [85], which is linked to key hepatic enzymes associated with animal metabolism and related biomarkers in liver tissue and plasma during the periparturient period (Table 3). Overall, higher plasma concentrations of fatty acids in high BCS compared with normal BCS cows were observed. Although there was a similar reactive oxygen metabolite (ROM) concentration across both groups, high BCS cows recorded lower overall concentrations of β-carotene and tocopherol, explaining the lower indicator (ferric reducing ability of plasma) of antioxidant capacity. High BCS cows had lower hepatic protein abundance of the 1-carbon metabolism enzymes cystathionine-β-synthase, betaine-homocysteine methyltransferase, and methionine adenosyltransferase 1 A (MAT1A), as well as the glutathione metabolism-related enzymes glutathione S-transferase α 4 and GPx3. A lower protein abundance of glutathione S-transferase mu 1 (GSTM1) at −15 days before calving and 7 days after calving was also observed. Regardless of BCS, cows featured increased abundance of GSTM1 and GPx3 between −15 and 7 days. A marked decrease of gamma-butyrobetaine dioxygenase 1 from −10 to 7 days in high BCS compared with normal BCS cows suggested a decrease in de novo carnitine synthesis that was partly explained by the lower abundance of MAT1A. Overall, the data suggest biological links between BCS before calving, milk yield, immune response, and hepatic reactions encompassing 1-carbon metabolism, carnitine, and antioxidant synthesis.

Loss of body condition during the dry period and early postpartum has been linked to an increased risk of cows contracting metabolic and infectious diseases after calving [86,87]. Cows with a higher BCS at the end of lactation invariably lose more condition during the pre-calving period, whereas under conditioned cows gain or sustain body condition during the dry period [87,88]. Numerous experts concur that reducing BCS in high-yielding dairy cows could mitigate the effect of NEB, one of the primary causes of suboptimal reproductive function [89]. Increased NEFA and BHBA blood levels, as well as decreased insulin and glucose levels, are all markers of NEB [90]. De Koster et al. (2015) [91] discovered that obese cows had a lower insulin sensitivity for glucose metabolism. Thus, increased NEFA and BHB levels, as well as decreased glucose and IGF-1 levels, are considered biomarkers of a failed transition from the dry to lactation period [92,93]. NEFA contributes to the generation of ROS, resulting in an imbalance of oxidative and antioxidant species, activation of p53 transcriptional activity, inhibition of Nrf2 transcriptional activity, loss of mitochondrial membrane potential (MMP), and release of apoptosis-inducing factor (AIF) and cytochrome c (cyt c) into the cytosol, finally leading to hepatocytes apoptosis [94,95]. Cows with higher BCS levels are more susceptible to oxidative stress [96,97].

**Table 2:** Summary of high concentrate feed effect on gene expression in pro and anti-inflammatory cytokines, chemokines, glucose metabolism, lipid metabolism, and protein expression of dairy cows.

Animals and Status	Treatment	Target	Gene Expression MAPK Pathway and Antioxidant Genes, Pro and Anti-Inflammatory Cytokines, Chemokines, Glucose and Lipid Metabolism, Protein Expression	References
Mid-lactation Cows:	LC diet with F:C of 6:4 HC diet with F:C of 4:6	Mammary epithelial tissue	SOD1, SOD2, GPx1 and GP13 genes expression decrease in HC diet group, no difference on SOD3 pJNK and pp38 expression was increased in LC ERK and pERK were significantly higher in HC diet ERK, p38 and JNK increased in HC diet group No difference in JNK and p38 pNrf2 and Nrf2 protein indicated a reduction in HC diet	[72]
Mid-lactation Cows:	LG diet with F:C of 6:4 HG diet with F:C of 4:6	Liver	Expression levels of target genes decreased in GPX1 and CAT in the HG The SOD1 was increased in cows fed HG NF-kB mRNA expression was increased in HG, Nrf2 mRNA expression was lower in the HG Nrf2 target genes, such as NQO1, MT1E, MGST3, MT1A and UGT1A1 were downregulated in HG Other Nrf2 target genes, such as TXNRD1, HMOX2, SRXN1 and MT2A were not affected by HG The expression levels of Nrf2 protein were decreased in the livers of cows fed HG	[73]
Mid-lactating Holstein cows:	LC diet with 40% grain, control HC diet with 60% grain	Mammary gland	NOD1 and RIP2 increased in HC group NF-κB, was significantly higher in HC group IL-6, IL-8, and TNF-α were up-regulated in HC group no difference in the expression level of IL-1β The protein expression of NOD1 was up-regulated in HC group The protein expression of p65 and pp65 increased in HC group,	[98]
Lactating Holstein dairy cows	LC diet with F:C of 6:4 HC diet with F:C of 4:6	Liver	TRAF6 NF-κB,ERK, MAPK, p38 mitogen activated protein kinase (MAPK) was increased by HC feeding No difference in mRNA expression of TLR-4 IL-1 and acute-phase protein serum amyloid A(SAA) increased by HC TRAF6, NF-κB, p38, ERK increased by HC feeding No difference in ERK MAPK phosphorylation level	[68]
Mid-lactating dairy cows	HC diet NFC:NDF; 6:4 LC diet NFC:NDF of 4:6	Uterus	gene expression of TLR4 and MyD88 in the HC group was upregulated TRAF-6 and NF-κB were highly expressed in HC group The gene expression of IL-1β, IL-6 and LBP was also higher in the HC group	[78]
Mid-lactating Holstein cows: (2 treatment groups)	LC diet with F:C 6:4 HC diet with F:C 4:6	Mammary gland	IL-1β, IL-6 and TNF-α, increased in the HC group in the lacteal vein the mRNA expression level of NF-κB, was higher in the HC group IL-6, IL-8, and TNF-α were up-regulated in the mammary gland of the HC group No difference in the expression level of IL1β Protein expression of NOD1 was up-regulated in the HC group The protein expression of p65 and pp65 increased in HC group	[99]
Mid-lactating Holstein cows: (2 treatment groups)	LC diet with F:C 6:4 HC diet with F:C 4:6	Mammary gland tissue	NOD1, Rip-2, Bax mRNA Caspase-3, Caspase-8, and Caspase-9 mRNA expressions were higher in HC group Bcl-2 mRNA expressions were lower in HC group NOD1, and Caspase-3 protein expressions and Caspase-8, and Bax expressions were higher in HC group Bcl-2 protein expressions were lower in HC group	[98]
Mid-lactating cows:	LC diet with F:C 6:4 HC diet with F:C 4:6	Mammary gland	MPO activity in the mammary tissue and the lacteal vein plasma in HC group was up-regulated The NAG activity in the lacteal vein plasma in HC group was increased The expression of LAP, IL-1β, IL-6, IL-8, and TNF-α increased in the HC group BNBD5 up-regulated tendency expression in HC group no difference in NF-kB LAP protein expression was up-regulated in the HC group	[100]

F:C: forage to concentrate ratio, HC: high concentrate, HG: high grain, LAP: Leucine Aminopeptidase, LBP: lipopolysaccharide binding protein, LC: low concentrate, LG: low grain, MAPK: mitogen-activated protein kinase, MPO: myeloperoxidase, NA: not available, NAG: β-*N*-acetyl glucosaminidase, NFC:NDF: non-forage carbohydrates:neutral detergent fiber, NF-κB: nuclear factor κB, SAA: serum amyloid A, SARA: subacute ruminal acidosis.

**Table 3:** Summary of body condition score (BCS) effect on milk, blood, and oxidative status of dairy cows.

Status	Treatment	Milk Yield, Milk FA and Secretion	Blood Metabolites/Hormones	Oxidative and Anti-Oxidative Biomarkers	References
Transition cows selected four weeks before calving	High BCS ≥ 3.50 Normal BCS ≤ 3.25	Overall milk production was increased in high BCS cows No differences in milk percentage but lactose was lower in high BCS cow	NEFA and BHBA were increased in high BCS cows	No difference in ROM concentrations Myeloperoxidase, MDA and SOD were greater in high BCS cows FRAP decrease in high BCS cows β-carotene and tocopherol were lower in high BCS cows	[101]
transition cows	Medium BCS 3.25–3.75 high BCS ≥ 4 (pre-calving)	milk production increased with high BCS No difference in milk fat percentage by BCS	Plasma glucose, insulin, triglycerides, and cholesterol concentration were not affected by high BCS NEFA and BHBA increased in high BCS	The concentrations of plasma albumin, MDA and TAC were not affected by high BCS Plasma SOD and GSHx activity were reduced at high BCS	[102]
transition cows: (prepartum)	Low BCS ≤ 2.5 Medium: 2.75 ≤ BCS ≤ 3.5) high (BCS ≥ 3.75)	No difference in Milk yield Milk fat content was highest for high-BCS Milk protein yield was lowest for the low-BCS Low BCS had greater milk content of 10:0, 12:0, 14:0, 15:0, 16:0, 20:0, 20:3n − 6, 22:0, ∑SFA, ∑BCFA, and ∑10:0 to 15:0 High BCS had greater milk content of cis-9 18:1, ∑cis 18:1, cis-9,cis-12 18:2, cis-9, trans-11 CLA, cis-11 20:1, ∑MUFA, and ∑PUFA The medium-BCS group had the greatest concentrations of 18:3n − 3 and cis-9 20:1	No difference in plasma glucose and insulin Plasma NEFA and BHBA were greatest in high-BCS Plasma IGF-1 and leptin concentrations were greatest for the high-BCS group	NA	[103]
Lactating cows	Low BCS BCS ≤ 2.75 Medium BCS 3.0–3.5High BCS BCS ≥ 3.75	NA	The serum insulin, NEFA, and Triglyceride in high-BCS cows was increased RQUICKI and VLDL decreased in high BCS Serum apo A-I and apo B were higher in low-BCS and mid BCS cows No difference in glucose, glucagon, cholesterol and HDL-C and BHBA	Albumin and ceruloplasmin were lower in low BCS cows The serum ROS of mid-BCS and low BCS cows was higher Serum gamma-glutamyl transpeptidase, aspartate aminotransferase, alkaline phosphatase, bilirubin, SOD, GSH-Px, PON, myeloperoxidase, and MDA were not affected by BCS	[104]
transition cows	Cows with low BCS < 2.5 Cows with mid BCS 2.6 to 3.0 Cows with high BCS > 3.0	NA	No difference in plasma glucose High-BCS cows had higher plasma BHBA and NEFA	High-BCS group showed higher plasma ROM, TBARS and SH; lower erythrocyte SH and SOD No differences in plasma and erythrocyte GSH-Px No difference in the oxidative status	[96]

BCS: body condition score, FRAP: ferric reducing ability of plasma, ROS: reactive oxygen species, ROM: reactive oxygen metabolite, SOD: superoxide dismutase, MDA: malondialdehyde, GSH-Px: glutathione peroxidase, PON: paraoxonase, SH: thiol groups, TBARS: thiobarbituric acid-reactive substances; HDL-C: high-density lipoprotein cholesterol; ROS: reactive oxygen species, RQUICKI: revised quantitative insulin sensitivity check index, VLDL: very low-density lipoprotein, SFA: saturated fatty acid, BCFA: branched-chain fatty acids, MUFA: monounsaturated fatty acid, NA: not available, PUFA: polyunsaturated fatty acid, CLA: conjugated linoleic acid, IGF: insulin-like growth factor 1.

## 4. The Role of Specific Feed Additives Alleviating Oxidative Stress

Efficient management of oxidative stress comes from understanding roles of specific functional elements in the diet. Amino acids, vitamins, trace elements, and plant extracts containing a diversity of metabolites are known in traditional Chinese medicine for their anti-inflammatory and anti-oxidative values. Therefore, those functional feed additives need to be continuously valorized in animal nutrition to mitigate oxidative stress cases, especially when natural animal antioxidant defenses are overwhelmed.

### 4.1. Rumen Protected Amino-Acids: Methionine and Lysine, L-arginine, and N-carbamylglutamate

Methionine and lysine are the building blocks of casein synthesis, but they are unfortunately also known as limiting amino acids (AA) for milk protein metabolism in ruminants, while casein derivatives mediate anti-oxidative actions [105]. Studies on the effects of dietary inclusion of rumen protected methionine and lysine on oxidative status [106], immune response reproduction, and milk performance [107] of dairy cattle have been well documented, but limiting AA have been tested as immune function regulators in dairy cows during early lactation [108]. Moreover, little information exists on optimum inclusion levels and synergetic effects of rumen protected amino-acids on their oxidative status Mavrommatis et al. (2021) [109] recently found that a decrease in plasma BHBA led to increased glutathione peroxidase (GSH-Px) activity in lactating ewes that were provided a combination of rumen protected methionine and lysine. Therefore, increasing antioxidants activity has a beneficial effect on animal health and can decrease the incidence rate of metabolic disorder diseases like ketosis. Furthermore, AA contribute to cellular oxidative balance [110] through participating in taurine and GSH synthesis [111], ensuring cellular detoxification and hydrogen peroxide neutralization via glutathione S-transferase and GSH-Px actions, respectively. Methionine plays a key role in de novo short- and medium-chain FA synthesis as a source of methyl for the transmethylation reactions in lipids biosynthesis [112]. It also enhanced de novo glutathione and carnitine synthesis in the liver, and thus increased antioxidant and β-oxidation capacity [113]. Sun et al. [114] reported that supplementation of methionine increase the “very-low-density lipoproteins” (VLDL) resulting in enhanced vitamin E circulation. He also demonstrated that dietary supplementation of rumen protected amino-acids can suppress side effects of lipid peroxidation by-products such as MDA. Moreover, higher FRAP values in blood plasma and milk of ewes fed combinations of rumen-protected Met + choline + betaine compared with the control were observed [115]. Besides the inclusion of methionine and lysine and its benefit in enhancing antioxidants status, *N*-carbamylglutamate (NCG, a metabolically stable analogue of *N*-acetylglutamate synthase (NAG) that produces arginine endogenously) can also play a significant role in improving immune function and oxidative status in suckling lambs. Zhang et al. (2018) [116] conducted a study to investigate effects of dietary supplementation with l-arginine (Arg) and *N*-carbamylglutamate (NCG) on intrauterine growth-retarded (IUGR) suckling lambs. They showed that the concentrations of protein carbonyl (PCs) and MDA were lower and the glutathione (GSH) concentration and ratio of GSH/GSSG greater in the jejunum, duodenum and ileum of IUGR + 1% Arg or 0.1% NCG lambs, compared to IUGR group. Zhou et al. (2016) [117] isolated polymorphonuclear leukocytes (PMNL) and showed the lower abundance of genes linked to inflammation (IL1B, TLR2, NF-κB, and STAT3) and oxidative stress (CBS, GPx1, glutathione synthase [GSS], and SOD2) as well as an increase in plasma taurine with methionine provision, and proposed improved redox and inflammatory status of those cells. A recent trial conducted by Lopreiato et al. (2019) [118] studied the consequences of incubation bovine PMNL with Met and/or choline and observed that methionine supplementation coupled with sufficient choline enhanced gene expression of TLR2 and l-selectin, which are pathogen recognition mechanisms. In the same trial, cells incubated without choline had high mRNA abundances encoding IL1B, IL6, IL10, and myeloperoxidase (MPO), glutathione reductase (GSR), GSS, cystathionine gamma-lyase (CTH), and cysteine sulfinic acid decarboxylase (CSAD), suggesting higher inflammation and oxidative stress.

### 4.2. Vitamins, Trace Elements and Plant Extracts

A daily supplementation of 1000 IU vitamin E in diet of dairy cows significantly reduced stress markers such as MDA and heat shock protein 70 and increased activities of SOD and GSH-Px [119]. Moreover, serum immunoglobulin and interleukin concentrations increased significantly, and the activities of T-AOC and various antioxidant enzymes increased in dairy cows supplementing typical lactation diets with 110 and 220 IU/kg of vitamin A. Supplementing vitamin E (80 IU/kg) and selenium (5 mg/kg) in the last gestation month increased the serum levels of the mineral in the cows, improved the reproductive performance, and reduced incidents of sub-clinics mastitis [120].

Trace elements such as copper (Cu), manganese (Mn), zinc (Zn), and selenium (Se) can also improve antioxidant functions in dairy cows. Se is involved in the synthesis of GSH-Px [121]. An adequate selenium status is essential for many antioxidant processes. Sun et al., (2019) [122] reported that 0.3 mg/kg DM hydroxy-selenomethionine (HMSeBA) decreases some parameters (e.g., NO, MDA) of heat stress-induced oxidative stress. The supplementation of HMSeBA (0.1, 0.3, or 0.5 mg of Se/kg of DM) linearly increased the activities of serum GSH-Px and SOD, but decreased MDA content [123]. Lower stress levels and higher immune response were observed when 60 ppm Zn were supplemented to the TMR diet of healthy multiparous cows [124].

Resveratrol is a natural polyphenol present in plants such as grapes, blueberries, and mulberries. Many studies have reported that resveratrol can exert antioxidant effects. Zhou et al. (2019) [125] reported that resveratrol alleviates aflatoxin B1-induced cytotoxicity, including the increase in ROS and the decrease in mitochondrial membrane potential (MMP) and apoptosis in MAC-T cow mammary epithelial cell line. With Resveratrol, MAC-T cells avoided ROS H_2_O_2_-induced endoplasmic reticulum stress and mitochondria-related cell apoptosis. Moreover, resveratrol induced mRNA expression of multiple antioxidant defense genes in MAC-T cells under normal/oxidative conditions [100]. Daidzein, an isoflavone extract with phytoestrogenic properties, can regulate specific and non-specific immune functions in animals through an endocrine system regulation [126]. Liu et al. (2014) [127] reported that 300 and 400 mg/day daidzein treatment increased IgG and IL-2 in serum of late lactation cows. Therefore, daidzein can enhance immuno-competence of late lactation cows and strengthen their resistance to heat stress.

## 5. Antioxidant Mechanisms of Action to Regulate Oxidative Stress

Studies found that nuclear factor E2-related factor 2-antioxidant response element (Nrf2-ARE), NF-κB, MAPK and phosphatidylinositol-3-kinase (PI3K)/protein kinase B (Akt) are in an important signal transduction pathway through which ingested feed additives exert antioxidant capacity and reduce oxidative stress (Table 4).

### 5.1. Nrf2-ARE Signal Transduction Pathway

The activity of Nrf2 is mainly regulated by Kelch-like epichlorohydrin-related protein-1. Under normal circumstances, Kelch-like epichlorohydrin-related protein-1 binds to actin, anchoring Nrf2 in the cytoplasm, and acts as an endogenous antioxidant. In case of oxidative stress, the Nrf2-ARE signal transduction pathway is activated, and the conformation of Kelch-like epichlorohydrin-related protein-1 changes to release Nrf2 into the nucleus which will promote, in combination with ARE, a variety of antioxidants including a phase II detoxification enzyme expression, all of which play protective roles related to cell oxidative stress damage [128,129].

### 5.2. NF-κB Signal Transduction Pathway

When the cell is not subjected to related stimulation, NF-κB exists in two forms in the cytoplasm. One is that the p65 subunit of NF-κB binds to the IκB protein, which can cover the nuclear localization signal (NLS) of p50, so that NF-κB and IκB form a trimer. When cells are subjected to appropriate extracellular signals that activate NF-κB, they mainly include inflammatory cytokines (such as IL-1, TNF-α), growth factors, and immune receptors (such as CD40, FasL), neurotoxins, bacteria, and their metabolites LPS, and viruses. When multiple factors such as viral proteins and certain physical and chemical factors (such as ultraviolet light o oxygen free radicals) are stimulated, they cause a series of enzymatic chain reactions [133,134].

### 5.3. MAPK Signal Transduction Pathway

MAPK family includes p38MAPK, JNK, and extracellular signal-regulated kinase 1/2 (ERK1/2), all of which are closely related to intracellular oxidative reduction responses [135]. The activation of activator protein 1 (AP-1) caused by H202, cytokines and other stress factors is mainly regulated by JNK and p38MAPK pathways. The activation of the pathways further increases the release of inflammatory factors, such as IL-lp, IL-6, and TNF-a [136,137]. The apoptosis signal—regulating kinase 1 (ASK-1)—is an intermediate of the MAPK signaling pathway, which can activate downstream pro-inflammatory and pro-apoptotic cell-signaling cascade [138].

## 6. Future Directions

In recent years, the development and scale of the dairy farming industry has grown, and improvement of its economic efficiency remains a high priority. Unfortunately, dairy cows experience various oxidative stress effects from calving to the peak of lactation. Alleviative measures to meet the nutritional challenges of food security need to be further developed. Therefore, supplementing the appropriate amount of vitamins, trace elements, and plant extracts in the animal’s diet can effectively improve the oxidative stress of dairy cows across a variety of signal transduction pathways aimed at preventing damage and repairing damaged cells. Therefore, the mechanisms of exogenous antioxidants need to be further studied and clarified. The fundamental pathways and mechanisms examined here will be critical to that endeavor.

## Figures and Tables

**Table 1:** Summary of high concentrate feed effects on milk, blood, and oxidative status of dairy cows.

Status	Treatment	Milk Yield and Composition	Milk and Blood Metabolites, Enzymes and Hormones	Oxidative and Anti-Oxidative Biomarkers	References
Mid-lactation cows:	LC diet with F:C of 6:4, DM HC diet with F:C of 4:6, DM	NA	NA	LPS level in the rumen fluid, plasma, mammary glands increased in HC ROS and MDA increased in HC T-AOC enzyme decreased in HC	[72]
Mid-lactation cows:	LG diet with F:C of 6:4 HG diet with F:C of 4:6, DM	Milk yield, percentage of milk fat, and the milk fat yield decreased in cows fed HG The percentage of milk protein increased in HG group	NA	LPS in the rumen fluid, hepatic, portal and jugular vein plasma were higher in the cows fed HG MDA and SOD activity increased in HG cows T-AOC, GPx, and catalase activity was reduced in HG cows Total NOS and iNOS activity in the liver and plasma increase in HG	[73]
Lactating Holstein dairy cows:	HC diet with F:C of 4:6 LC diet with F:C of 6:4	NA	Aminotransferase (AST), alanine aminotransferase (ALT) and lactate dehydrogenase (LDH) activity increased in HC group While, albumin (ALB) and total protein (TP) concentrations in the peripheral blood of the HC group were reduced	The LPS levels in portal vein and hepatic vein increased in HC group	[74]
Early-lactating Simmental cows:	40% concentrate: control diet 60% concentrate to induce SARA	NA	SARA cows showed higher plasma glucose and AST concentrations The concentration of lactate in blood was higher in cows that received the SARA-feeding No difference in NEFA, cholesterol, GLDH concentrations The concentration of BHBA and gamma-glutamyltransferase (GGT) were higher in control cows	NA	[75]
late-lactation Holstein cows:	F:C diet of 50:50 without buffer (MCNB) F:C diet of 50:50 with buffer (MCWB) F:C diet of 25:75 without buffer (HCNB) F:C diet of 25:75 with buffer (HCWB)	Milk yield and Milk lactose did not change Milk fat percentage decreased as the amount of concentrate increased Milk crude protein percentage increased as the amount of concentrate included in the diet increased	Total short, medium, and long-chain FA and total monounsaturated, polyunsaturated, and saturated FA remain unchanged Concentrations of C14:1, cis-∆-9-C16:1, and total-C16:1 increased, whereas C17:0, C18:0, C18:3, C20:0, and C24:0 decreased with an increased level of concentrate in the diet The milk trans-∆-10,11 C18:1 FA concentrations of animals fed the HCNB was increased	NA	[76]
Lactating cows:	LFC: with F:C of 55:45 HFC: with F:C of 70:30	NA	AST and creatine kinase were higher in the HFC No difference in ALT, GGT, ALP, and LDH	Reactive oxygen metabolites (d-ROMs) were lower in the HFC group No differences for antioxidant barrier (Oxy-adsorbent tests) and anti ROMs	[77]
Mid-lactating dairy cows:	HC diet with NFC:NDF; 6:4 LC diet with NDF: NFC; 4:6	NA	NA	The LPS concentrations in the rumen fluid and the jugular vein plasma were significantly higher in the cows fed an HC diet	[78]
Mid-lactating cows:	HC diet with F:C of 4:6 LC diet with F:C of 6:4	Milk yields increased after 4 wks and decreased after 6 wks in HC	NA	LPS in the rumen, portal, and hepatic veins were higher in the high concentrate group	[79]

ALP: alkaline phosphatase, ALT: alanine aminotransferase, AST: aspartate aminotransferase, BHBA: β-hydroxybutyrate, CLA: conjugated linoleic acid, DM: dry matter, FA: fatty acid, F:C: forage to concentrate ratio, GGT: gamma-glutamyltransferase, GLDH: Glutamate dehydrogenase, GPx: glutathione peroxidase, HC: high concentrate, HCNB: concentrate to forage 75:25 without buffer, HCWB: concentrate to forage 75:25 with buffer, HFC: high forage concentration, HG: high grain, iNOS: induction of nitric oxide synthase, LBP: lipopolysaccharide binding protein, LC: low concentrate, LDH: Lactate dehydrogenase, LFC: low forage concentration, LG: low grain, LPS: lipopolysaccharide, MCNB: concentrate to forage 50:50 without buffer, MCWB: concentrate to forage 50:50 with buffer, MDA: malondialdehyde, NA: not available, NEFA: non-esterified fatty acids, NFC:NDF: non-forage carbohydrates: neutral detergent fiber, NOS: nitric oxide synthase, ROM: reactive oxygen metabolites, ROS: reactive oxygen species, SARA: subacute ruminal acidosis, SOD: superoxide dismutase, T-OAC: total antioxidant capacity. Increased imbalance of redox status has been observed in high-yielding dairy cows during early lactation, when starch content increases in diets. This may be due to cellular changes associated with oxidative phosphorylation [80].

**Table 4:** Mechanism of action of specific dietary interventions to mitigate oxidative stress in dairy cows.

Dietary Intervention	Effects	Mode of Action	References
Control and RPAA RPAA: L, LML, HML, M	Methionine improves the organism’s OS status, without adversely affecting milk’s oxidative stability. Lysine affects negatively the milk’s oxidative stability. Milk’s fat increase with RPAA	M, LML, HML reduce plasma MDA, M, LML increase plasma GST activity; LML and HML reduce FRAP; LML reduces plasma ABTS; Lysine increased milk’s FRAP, MDA; L, HML diets increased milk’s protein carbonyls	[109]
0.2 g/kg DM green tea polyphenol	Improves the milk yield and health status in cows with hyperketonemia	Improve immune and antioxidant functions of dairy cows, decrease oxidative stress level	[130]
105 IU Vitamin A, 60ppm zinc and 2500IU vitamin E	Higher immune response	Decrease the somatic cell count of the milk	[124]
220 IU/kg Vitamin A	Improve immune and antioxidant functions of dairy cows	Decrease the somatic cell count of the milk	[125]
1000 IU Vitamin E	Improve reproductive performance of Holstein Frisian and crossbred cows	Decrease plasma MDA, HSP-70 and cortisol; increase SOD and GPx activities	[131]
0.1, 0.3, or 0.5 mg of HMSeBA /kg	Decrease oxidative stress of dairy cows	Increased the activities of serum GPx and SOD	[122]
200, 300, 400 mg/day daidzein	Strengthen cow resistance to heat stress	Increase IgG, interferon alpha (IFN-), and interleukin-2 (IL-2)	[127]
25.79, 51.58, 103.16, and 154.74 mg/day zinc oxide	Improve antioxidant status of newborn calves	Improve zinc metabolism	[132]

C: basal diet (control); RPAA: Rumen protected amino-acid; M: basal diet + 6 g/ewe RP methionine; L: basal diet + 5 g/ewe RP lysine; LML: basal diet + 6 g methionine and 5 g lysine/ewe; and HML: basal diet + 12 g methionine + 5 g lysine/ewe. HMSeBA: hydroxy-selenomethionine, SOD: superoxide dismutase, GSH-Px: glutathione peroxidase, GST: glutathione transferase, IgG: immunoglobulin G, MDA: malondialdehyde, HSP-70: Heat shock protein-70; FRAP: ferric reducing ability of plasma, ABTS: 2,20-Azino-bis 3-ethylbenzthiazoline-6-sulfonic acid.

## References

[1] Benov L. (2001). How Superoxide Radical Damages the Cell. Protoplasma.

[2] Halliwell B. (1978). Superoxide-dependant formation of hydroxyl radicals in the presence of iron chelates: Is it a mechanism for hydroxyl radical production in biological systems?. FEBS Lett..

[3] McCord J.M., Day E.D. (1978). Superoxide-dependant production of hydroxyl radical catalyzed by iron-EDTA complex. FEBS Lett..

[4] Rendra E., Riabov V., Mossel D.M., Sevastyanova T., Harmsen M.C., Kzhyshkowska J. (2019). Reactive Oxygen Species (ROS) in Macrophage Activation and Function in Diabetes. Immunobiology.

[5] Suzuki Y. (2019). Oxidant-Mediated Protein Amino Acid Conversion. Antioxidants.

[6] Martinelli I., Tomassoni D., Roy P., Di Cesare Mannelli L., Amenta F., Tayebati S.K. (2021). Antioxidant Properties of Alpha-Lipoic (Thioctic) Acid Treatment on Renal and Heart Parenchyma in a Rat Model of Hypertension. Antioxidants.

[7] Orrenius S. (1985). Oxidative Stress Studied in Intact Mammalian Cells. Philos. Trans. R. Soc. B Biol. Sci..

[8] Riley P.A. (1994). Free Radicals in Biology: Oxidative Stress and the Effects of Ionizing Radiation. Int. J. Radiat. Biol..

[9] Behairy A., El-Sharkawy N.I., Saber T.M., Soliman M.M., Metwally M.M.M., Abd El-Rahman G.I., Abd-Elhakim Y.M., El Deib M.M. (2020). The Modulatory Role of Vitamin C in Boldenone Undecylenate Induced Testicular Oxidative Damage and Androgen Receptor Dysregulation in Adult Male Rats. Antioxidants.

[10] Nemec Svete A., Vovk T., Bohar Topolovec M., Kruljc P. (2021). Effects of Vitamin E and Coenzyme Q10 Supplementation on Oxidative Stress Parameters in Untrained Leisure Horses Subjected to Acute Moderate Exercise. Antioxidants.

[11] Hansen J.M., Go Y.-M., Jones D.P. (2006). Nuclear and Mitochondrial Compartmentation of Oxidative Stress and Redox Signaling. Annu. Rev. Pharm. Toxicol..

[12] Jové M., Mota-Martorell N., Pradas I., Martin-Gari M., Ayala V., Pamplona R. (2020). The Advanced Lipoxidation End-Product Malondialdehyde-Lysine in Aging and Longevity. Antioxidants.

[13] Awawdeh M.S. (2016). Rumen-Protected Methionine and Lysine: Effects on Milk Production and Plasma Amino Acids of Dairy Cows with Reference to Metabolisable Protein Status. J. Dairy Res..

[14] Martinov M.V., Vitvitsky V.M., Banerjee R., Ataullakhanov. F.I. (2010). The logic of the hepatic methionine metabolic cycle. Biochim. Biophys. Acta - Proteins Proteom..

[15] Seegers H., Fourichon C., Beaudeau F. (2003). Production Effects Related to Mastitis and Mastitis Economics in Dairy Cattle Herds. Vet. Res..

[16] Halasa T., Huijps K., Østerås O., Hogeveen H. (2007). Economic Effects of Bovine Mastitis and Mastitis Management: A Review. Vet. Q..

[17] Xue M., Sun H., Wu X., Guan L.L., Liu J. (2018). Assessment of Rumen Microbiota from a Large Dairy Cattle Cohort Reveals the Pan and Core Bacteriomes Contributing to Varied Phenotypes. Appl. Environ. Microbiol..

[18] Sørensen L.P., Mark T., Madsen P., Lund M.S. (2009). Genetic Correlations between Pathogen-Specific Mastitis and Somatic Cell Count in Danish Holsteins. J. Dairy Sci..

[19] Forbes D., Gehm W. (2011). Bacterial Migration through Teat Canal Related to Liner Action. Udder Health and Communication.

[20] Latia Mastitis in Dairy Cattle. Press Release 2017. https://www.latiaagribusinesssolutions.com/2017/10/04/mastitis-dairy-cattle/.

[21] Bradley A.J., Green M.J. (2001). Adaptation of Escherichia Coli to the Bovine Mammary Gland. J. Clin. Microbiol..

[22] Bhatt V.D., Ahir V.B., Koringa P.G., Jakhesara S.J., Rank D.N., Nauriyal D.S., Kunjadia A.P., Joshi C.G. (2012). Milk Microbiome Signatures of Subclinical Mastitis-Affected Cattle Analysed by Shotgun Sequencing. J. Appl. Microbiol..

[23] Falentin H., Rault L., Nicolas A., Bouchard D.S., Lassalas J., Lamberton P., Aubry J.-M., Marnet P.-G., Le Loir Y., Even S. (2016). Bovine Teat Microbiome Analysis Revealed Reduced Alpha Diversity and Significant Changes in Taxonomic Profiles in Quarters with a History of Mastitis. Front. Microbiol..

[24] Tong J., Zhang H., Zhang Y., Xiong B., Jiang L. (2019). Microbiome and Metabolome Analyses of Milk From Dairy Cows With Subclinical Streptococcus Agalactiae Mastitis—Potential Biomarkers. Front. Microbiol..

[25] Sharma N., Singh N.K., Bhadwal M.S. (2011). Relationship of Somatic Cell Count and Mastitis: An Overview. Asian-Australas. J. Anim. Sci..

[26] de Haas Y., Barkema H.W., Veerkamp R.F. (2002). The Effect of Pathogen-Specific Clinical Mastitis on the Lactation Curve for Somatic Cell Count. J. Dairy Sci..

[27] Smith K.L., Hogan J.S. (1993). Environmental mastitis. Vet. Clin. North Am. Food Anim. Pract..

[28] Fox L.K., Gay J.M. (1993). Contagious mastitis. Vet. Clin. North Am. Food Anim. Pract..

[29] Wilson C.D., Richards M.S. (1998). A survey of mastitis in the British dairy herd. Vet. Rec..

[30] Almeida R.A., Matthews K.R., Cifrian E., Guidry A.J., Oliver S.P. (1996). *Staphylococcus aureus* Invasion of Bovine Mammary Epithelial Cells. J. Dairy Sci..

[31] Baselga R., Albizu I., Amorena B. (1994). *Staphylococcus aureus* Capsule and Slime as Virulence Factors in Ruminant Mastitis. A Review. Vet. Microbiol..

[32] Jeon S.J., Cunha F., Ma X., Martinez N., Vieira-Neto A., Daetz R., Bicalho R.C., Lima S., Santos J.E.P., Jeong K.C. (2016). Uterine Microbiota and Immune Parameters Associated with Fever in Dairy Cows with Metritis. PLoS ONE.

[33] Bicalho M.L.S., Machado V.S., Higgins C.H., Lima F.S., Bicalho R.C. (2017). Genetic and Functional Analysis of the Bovine Uterine Microbiota. Part I: Metritis versus Healthy Cows. J. Dairy Sci..

[34] Jeon S.J., Lima F.S., Vieira-Neto A., Machado V.S., Lima S.F., Bicalho R.C., Santos J.E.P., Galvão K.N. (2018). Shift of Uterine Microbiota Associated with Antibiotic Treatment and Cure of Metritis in Dairy Cows. Vet. Microbiol..

[35] Hajishengallis G., Darveau R.P., Curtis M.A. (2012). The Keystone-Pathogen Hypothesis. Nat. Rev. Microbiol..

[36] Jeon S.J., Cunha F., Daetz R., Bicalho R.C., Lima S., Galvão K.N. (2021). Ceftiofur Reduced Fusobacterium Leading to Uterine Microbiota Alteration in Dairy Cows with Metritis. Anim. Microbiome.

[37] Antiabong J.F., Ball A.S., Brown M.H. (2015). The Effects of Iron Limitation and Cell Density on Prokaryotic Metabolism and Gene Expression: Excerpts from Fusobacterium Necrophorum Strain 774 (Sheep Isolate). Gene.

[38] Pérez-Báez J., Risco C.A., Chebel R.C., Gomes G.C., Greco L.F., Tao S., Thompson I.M., do Amaral B.C., Zenobi M.G., Martinez N. (2019). Association of Dry Matter Intake and Energy Balance Prepartum and Postpartum with Health Disorders Postpartum: Part I. Calving Disorders and Metritis. J. Dairy Sci..

[39] Galvão K.N., Flaminio M.J.B.F., Brittin S.B., Sper R., Fraga M., Caixeta L., Ricci A., Guard C.L., Butler W.R., Gilbert R.O. (2010). Association between Uterine Disease and Indicators of Neutrophil and Systemic Energy Status in Lactating Holstein Cows. J. Dairy Sci..

[40] Reynolds C.K., Aikman P.C., Lupoli B., Humphries D.J., Beever D.E. (2003). Splanchnic Metabolism of Dairy Cows during the Transition from Late Gestation through Early Lactation. J. Dairy Sci..

[41] Tsuchiya Y., Zhyvoloup A., Baković J., Thomas N., Yu B.Y.K., Das S., Orengo C., Newell C., Ward J., Saladino G. (2018). Protein CoAlation and Antioxidant Function of Coenzyme A in Prokaryotic Cells. Biochem. J..

[42] Castillo C., Pereira V., Abuelo Á., Hernández J. (2013). Effect of Supplementation with Antioxidants on the Quality of Bovine Milk and Meat Production. Sci. World J..

[43] Konvičná J., Vargová M., Paulíková I., Kovcáč G., Kostecká Z. (2015). Oxidative Stress and Antioxidant Status in Dairy Cows during Prepartal and Postpartal Periods. Acta Vet. Brno.

[44] Mòdol T., Brice N., Ruiz de Galarreta M., García Garzón A., Iraburu M.J., Martínez-Irujo J.J., López-Zabalza M.J. (2015). Fibronectin Peptides as Potential Regulators of Hepatic Fibrosis through Apoptosis of Hepatic Stellate Cells. J. Cell. Physiol..

[45] Berlier J.L., Rigutto S., Dalla Valle A., Lechanteur J., Soyfoo M.S., Gangji V., Rasschaert J. (2015). Adenosine Triphosphate Prevents Serum Deprivation-Induced Apoptosis in Human Mesenchymal Stem Cells via Activation of the MAPK Signaling Pathways. Stem Cells.

[46] Dröge W. (2002). Free Radicals in the Physiological Control of Cell Function. Physiol. Rev..

[47] Lykkesfeldt J., Svendsen O. (2007). Oxidants and Antioxidants in Disease: Oxidative Stress in Farm Animals. Vet. J..

[48] Chauhan S.S., Celi P., Ponnampalam E.N., Leury B.J., Liu F., Dunshea F.R. (2014). Antioxidant Dynamics in the Live Animal and Implications for Ruminant Health and Product (Meat/Milk) Quality: Role of Vitamin E and Selenium. Anim. Prod. Sci..

[49] Saquib Q., Al-Khedhairy A.A., Siddiqui M.A., Abou-Tarboush F.M., Azam A., Musarrat J. (2012). Titanium Dioxide Nanoparticles Induced Cytotoxicity, Oxidative Stress and DNA Damage in Human Amnion Epithelial (WISH) Cells. Toxicol. Vitr..

[50] Cheng G., Guo W., Han L., Chen E., Kong L., Wang L., Ai W., Song N., Li H., Chen H. (2013). Cerium Oxide Nanoparticles Induce Cytotoxicity in Human Hepatoma SMMC-7721 Cells via Oxidative Stress and the Activation of MAPK Signaling Pathways. Toxicol. Vitr..

[51] Abuelo A., Hernandez J., Benedito J.L., Castillo C. (2015). The importance of the oxidative status of dairy cattle in the periparturient period: Revisiting antioxidant supplementation. J. Anim. Physiol. Anim. Nutr..

[52] Piao D.C., Wang T., Lee J.S., Vega R.S., Kang S.K., Choi Y.J., Lee H.G. (2015). Determination of reference intervals for metabolic profile of Hanwoo cows at early, middle and late gestation periods. J. Anim. Sci. Biotechnol..

[53] Wang Y., Chun O.K., Song W.O. (2013). Plasma and dietary antioxidant status as cardiovascular disease risk factors: A review of human studies. Nutrients.

[54] Mertens D.R. (1997). Creating a system for meeting the fiber requirements of dairy cows. J. Dairy Sci..

[55] Kononoff P.J., Heinrichs A.J. (2002). Evaluating Particle Size of Forages and TMRs Using the New Penn State Forage Particle Separator.

[56] Humer E., Petri R.M., Aschenbach J.R., Bradford B.J., Penner G.B., Tafaj M., Südekum K.H., Zebeli Q. (2018). Invited review: Practical feeding management recommendations to mitigate the risk of subacute ruminal acidosis in dairy cattle. J. Dairy Sci..

[57] Chang G., Zhuang S., Seyfert H.M., Zhang K., Xu T., Jin D., Guo J., Shen X. (2015). Hepatic TLR4 signaling is activated by LPS from digestive tract during SARA, and epigenetic mechanisms contribute to enforced TLR4 expression. Oncotarget.

[58] Plaizier J.C., Krause D.O., Gozho G.N., McBride B.W. (2008). Subacute ruminal acidosis in dairy cows: The physiological causes, incidence and consequences. Vet. J..

[59] Stone W.C. (2004). Nutritional approaches to minimize subacute ruminal acidosis and laminitis in dairy cattle. J. Dairy Sci..

[60] Gozho G.N., Plaizier J.C., Krause D.O., Kennedy A.D., Wittenberg K.M. (2005). Subacute ruminal acidosis induces ruminal lipopolysaccharide endotoxin release and triggers an inflammatory response. J. Dairy Sci..

[61] Garrett E.F., Pereira M.N., Nordlund K.V., Armentano L.E., Goodger W.J., Oetzel G.R. (1999). Diagnostic methods for the detection of subacute ruminal acidosis in dairy cows. J. Dairy Sci..

[62] Kleen J.L., Upgang L., Rehage J. (2013). Prevalence and consequences of subacute ruminal acidosis in German dairy herds. Acta Vet. Scand..

[63] Emmanuel D.G., Madsen K.L., Churchill T.A., Dunn S.M., Ametaj B.N. (2007). Acidosis and lipopolysaccharide from Escherichia coli B:055 cause hyperpermeability of rumen and colon tissues. J. Dairy Sci..

[64] Gozho G.N., Krause D.O., Plaizier J.C. (2007). Ruminal lipopolysaccharide concentration and inflammatory response during grain-induced subacute ruminal acidosis in dairy cows. J. Dairy Sci..

[65] Khafipour E., Krause D.O., Plaizier J.C. (2009). A grain-based subacute ruminal acidosis challenge causes translocation of lipopolysaccharide and triggers inflammation. J. Dairy Sci..

[66] Zhang K., Chang G., Xu T., Xu L., Guo J., Jin D., Shen X. (2016). Lipopolysaccharide derived from the digestive tract activates inflammatory gene expression and inhibits casein synthesis in the mammary glands of lactating dairy cows. Oncotarget.

[67] Aviram M., Kaplan M., Rosenblat M., Fuhrman B. (2005). Dietary antioxidants and paraoxonases against LDL oxidation and atherosclerosis development. Handb. Exp. Pharm..

[68] Xu T., Tao H., Chang G., Zhang K., Xu L., Shen X. (2015). Lipopolysaccharide derived from the rumen down-regulates stearoyl-CoA desaturase 1 expression and alters fatty acid composition in the liver of dairy cows fed a high-concentrate diet. BMC Vet. Res..

[69] Zhao C., Liu G., Li X., Guan Y., Wang Y., Yuan X., Sun G., Wang Z., Li X. (2018). Inflammatory mechanism of Rumenitis in dairy cows with subacute ruminal acidosis. BMC Vet. Res..

[70] Sordillo L.M., Aitken S.L. (2009). Impact of oxidative stress on the health and immune function of dairy cattle. Vet. Immunol. Immunopathol..

[71] Sordillo L.M. (2018). Mammary Gland Immunobiology and Resistance to Mastitis. Vet. Clin. North Am. Food Anim. Pract..

[72] Memon M.A., Wang Y., Xu T., Ma N., Zhang H., Roy A.C., Aabdin Z.U., Shen X. (2019). Lipopolysaccharide induces oxidative stress by triggering MAPK and Nrf2 signalling pathways in mammary glands of dairy cows fed a high-concentrate diet. Microb. Pathog..

[73] Abaker J.A., Xu T.L., Jin D., Chang G.J., Zhang K., Shen X.Z. (2017). Lipopolysaccharide derived from the digestive tract provokes oxidative stress in the liver of dairy cows fed a high-grain diet. J. Dairy Sci..

[74] Guo J., Chang G., Zhang K., Xu L., Jin D., Bilal M.S., Shen X. (2017). Rumen-derived lipopolysaccharide provoked inflammatory injury in the liver of dairy cows fed a high-concentrate diet. Oncotarget.

[75] Aditya S., Humer E., Pourazad P., Khiaosa-Ard R., Zebeli Q. (2018). Metabolic and stress responses in dairy cows fed a concentrate-rich diet and submitted to intramammary lipopolysaccharide challenge. Animal.

[76] Khorasani G.R., Kennelly J.J. (2001). Influence of carbohydrate source and buffer on rumen fermentation characteristics, milk yield, and milk composition in late-lactation Holstein cows. J. Dairy Sci..

[77] Musco N., Tudisco R., Grossi M., Mastellone V., Morittu V.M., Pero M.E., Wanapat M., Trinchese G., Cavaliere G., Mollica M.P. (2020). Effect of a high forage: Concentrate ratio on milk yield, blood parameters and oxidative status in lactating cows. Anim. Prod. Sci..

[78] Bilal M.S., Abaker J.A., Ul Aabdin Z., Xu T., Dai H., Zhang K., Liu X., Shen X. (2016). Lipopolysaccharide derived from the digestive tract triggers an inflammatory response in the uterus of mid-lactating dairy cows during SARA. BMC Vet. Res..

[79] Chang G., Zhang K., Xu T., Jin D., Guo J., Zhuang S., Shen X. (2015). Epigenetic mechanisms contribute to the expression of immune related genes in the livers of dairy cows fed a high concentrate diet. PLoS ONE.

[80] Gabai G., Testoni S., Piccinini R., Marinelli L., Howard C.M., Stradaioli G. (2004). Oxidative stress in primiparous cows in relation to dietary starch and the progress of lactation. Anim. Sci..

[81] Roche J.R., Lee J.M., Macdonald K.A., Berry D.P. (2007). Relationships among body condition score, body weight, and milk production variables in pasture-based dairy cows. J. Dairy Sci..

[82] Barletta R.V., Maturana Filho M., Carvalho P.D., Del Valle T.A., Netto A.S., Rennó F.P., Mingoti R.D., Gandra J.R., Mourão G.B., Mourão G.B. (2017). Association of changes among body condition score during the transition period with NEFA and BHBA concentrations, milk production, fertility, and health of Holstein cows. Theriogenology.

[83] Kim I.H., Suh G.H. (2003). Effect of the amount of body condition loss from the dry to near calving periods on the subsequent body condition change, occurrence of postpartum diseases, metabolic parameters and reproductive performance in Holstein dairy cows. Theriogenology.

[84] Gärtner T., Gernand E., Gottschalk J., Donat K. (2019). Relationships between body condition, body condition loss, and serum metabolites during the transition period in primiparous and multiparous cows. J. Dairy Sci..

[85] Bewley J.M., Schutz M.M. (2008). An Interdisciplinary Review of Body Condition Scoring for Dairy Cattle. Prof. Anim. Sci..

[86] Kaufman E.I., LeBlanc S.J., McBride B.W., Duffield T.F., DeVries T.J. (2016). Short communication: Association of lying behavior and subclinical ketosis in transition dairy cows. J. Dairy Sci..

[87] Chebel R.C., Mendonça L.G.D., Baruselli P.S. (2018). Association between body condition score change during the dry period and postpartum health and performance. J. Dairy Sci..

[88] Daros R.R., Eriksson H.K., Weary D.M., von Keyserlingk M.A.G. (2020). The relationship between transition period diseases and lameness, feeding time, and body condition during the dry period. J. Dairy Sci..

[89] Stefańska B., Poźniak A., Nowak W. (2016). Relationship between the pre- and postpartum body condition scores and periparturient indices and fertility in high-yielding dairy cows. J. Vet. Res..

[90] Akbar H., Grala T.M., Vailati Riboni M., Cardoso F.C., Verkerk G., McGowan J., Macdonald K., Webster J., Schutz K., Meier S. (2015). Body condition score at calving affects systemic and hepatic transcriptome indicators of inflammation and nutrient metabolism in grazing dairy cows. J. Dairy Sci..

[91] De Koster J., Hostens M., Van Eetvelde M., Hermans K., Moerman S., Bogaert H., Depreester E., Van den Broeck W., Opsomer G. (2015). Insulin response of the glucose and fatty acid metabolism in dry dairy cows across a range of body condition scores. J. Dairy Sci..

[92] Ingvartsen K.L., Dewhurst R.J., Friggens N.C. (2003). On the relationship between lactational performance and health: Is it yield or metabolic imbalance that cause production diseases in dairy cattle? A position paper. Livest. Prod. Sci..

[93] Puppel K., Kuczyńska B. (2016). Metabolic profiles of cow’s blood; a review. J. Sci. Food Agric..

[94] Cui B.W., Bai T., Yang Y., Zhang Y., Jiang M., Yang H.X., Wu M., Liu J., Qiao C.Y., Zhan Z.Y. (2019). Thymoquinone Attenuates Acetaminophen Overdose-Induced Acute Liver Injury and Inflammation Via Regulation of JNK and AMPK Signaling Pathway. Am. J. Chin. Med..

[95] Li Y., Ding H., Liu L., Song Y., Du X., Feng S., Wang X., Li X., Wang Z., Li X. (2020). Non-esterified Fatty Acid Induce Dairy Cow Hepatocytes Apoptosis via the Mitochondria-Mediated ROS-JNK/ERK Signaling Pathway. Front. Cell. Dev. Biol..

[96] Bernabucci U., Ronchi B., Lacetera N., Nardone A. (2005). Influence of body condition score on relationships between metabolic status and oxidative stress in periparturient dairy cows. J. Dairy Sci..

[97] Laubenthal L., Ruda L., Sultana N., Winkler J., Rehage J., Meyer U., Dänicke S., Sauerwein H., Häussler S. (2017). Effect of increasing body condition on oxidative stress and mitochondrial biogenesis in subcutaneous adipose tissue depot of nonlactating dairy cows. J. Dairy Sci..

[98] Ul Aabdin Z., Cheng X., Dai H., Wang Y., Sahito B., Roy A.C., Memon M.A., Shen X. (2020). High-Concentrate Feeding to Dairy Cows Induces Apoptosis via the NOD1/Caspase-8 Pathway in Mammary Epithelial Cells. Genes.

[99] Wang Y., Zhang W., Ma N., Wang L., Dai H., Bilal M.S., Roy A.C., Shen X. (2019). Overfeeding with a high-concentrate diet activates the NOD1-NF-κB signalling pathway in the mammary gland of mid-lactating dairy cows. Microb. Pathog..

[100] Jin D., Chang G., Zhang K., Guo J., Xu T., Shen X. (2016). Rumen-derived lipopolysaccharide enhances the expression of lingual antimicrobial peptide in mammary glands of dairy cows fed a high-concentrate diet. BMC Vet. Res..

[101] Bucktrout R.E., Ma N., Aboragah A., Alharthi A.S., Liang Y., Lopreiato V., Lopes M.G., Trevisi E., Alhidary I.A., Fernandez C. (2021). One-carbon, carnitine, and glutathione metabolism-related biomarkers in peripartal Holstein cows are altered by prepartal body condition. J. Dairy Sci..

[102] Jamali Emam Gheise N., Riasi A., Zare Shahneh A., Celi P., Ghoreishi S.M. (2017). Effect of pre-calving body condition score and previous lactation on BCS change, blood metabolites, oxidative stress and milk production in Holstein dairy cows. Ital. J. Anim. Sci..

[103] Pires J.A., Delavaud C., Faulconnier Y., Pomiès D., Chilliard Y. (2013). Effects of body condition score at calving on indicators of fat and protein mobilization of periparturient Holstein-Friesian cows. J. Dairy Sci..

[104] Wu J., Liu J., Wang D. (2020). Effects of body condition on the insulin resistance, lipid metabolism and oxidative stress of lactating dairy cows. Lipids. Health Dis..

[105] Park J.K., Yeo J.M., Bae G.S., Kim E.J., Kim C.H. (2020). Effects of supplementing limiting amino acids on milk production in dairy cows consuming a corn grain and soybean meal-based diet. J. Anim. Sci. Technol..

[106] Coleman D.N., Lopreiato V., Alharthi A., Loor J.J. (2020). Amino acids and the regulation of oxidative stress and immune function in dairy cattle. J. Anim. Sci..

[107] Lopes M.G., Dominguez J.H.E., Corrêa M.N., Schmitt E., Fischer G. (2019). Rumen-protected methionine in cattle: Influences on reproduction, immune response, and productive performance. Arq. Inst. Biol..

[108] Tsiplakou E., Mavrommatis A., Skliros D., Righi F., Flemetakis E. (2020). The impact of rumen-protected amino acids on the expression of key-genes involved in the innate immunity of dairy sheep. PLoS ONE.

[109] Mavrommatis A., Giamouri E., Tavrizelou S., Zacharioudaki M., Danezis G., Simitzis P.E., Zoidis E., Tsiplakou E., Pappas A.C., Georgiou C.A. (2021). Impact of Mycotoxins on Animals’ Oxidative Status. Antioxidants.

[110] Yin J., Li T., Yin Y. (2016). Methionine and Antioxidant Potential. J. Antioxid. Act..

[111] Li P., Yin Y.L., Li D., Kim S.W., Wu G. (2007). Amino acids and immune function. Br. J. Nutr..

[112] Tsiplakou E., Mavrommatis A., Kalogeropoulos T., Chatzikonstantinou M., Koutsouli P., Sotirakoglou K., Labrou N., Zervas G. (2017). The effect of dietary supplementation with rumen-protected methionine alone or in combination with rumen-protected choline and betaine on sheep milk and antioxidant capacity. J. Anim. Physiol. Anim. Nutr..

[113] Osorio J.S., Ji P., Drackley J.K., Luchini D., Loor J.J. (2013). Supplemental Smartamine M or MetaSmart during the transition period benefits postpartal cow performance and blood neutrophil function. J. Dairy Sci..

[114] Sun F., Cao Y., Cai C., Li S., Yu C., Yao J. (2016). Regulation of Nutritional Metabolism in Transition Dairy Cows: Energy Homeostasis and Health in Response to Post-Ruminal Choline and Methionine. PLoS ONE.

[115] Zhang H., Zhao F., Peng A., Dong L., Wang M., Yu L., Loor J.J., Wang H. (2018). Effects of Dietary l-Arginine and N-Carbamylglutamate Supplementation on Intestinal Integrity, Immune Function, and Oxidative Status in Intrauterine-Growth-Retarded Suckling Lambs. J. Agric. Food Chem..

[116] Zhou Z., Loor J.J., Piccioli-Cappelli F., Librandi F., Lobley G.E., Trevisi E. (2016). Circulating amino acids in blood plasma during the peripartal period in dairy cows with different liver functionality index. J. Dairy Sci..

[117] Lopreiato V., Vailati-Riboni M., Bellingeri A., Khan I., Farina G., Parys C., Loor J.J. (2019). Inflammation and oxidative stress transcription profiles due to in vitro supply of methionine with or without choline in unstimulated blood polymorphonuclear leukocytes from lactating Holstein cows. J. Dairy Sci..

[118] Dong Z.W., Wang J.K., Liu J.X. (2014). Effects of Vitamin E on Performance of Dairy Cow as Antioxidant. Chin. J. Anim. Sci..

[119] Zanetti M.H. (1998). Effect of selenium and vitamin E supplementation in dairy cows. Rev. Bras. Zootecn..

[120] Rotruck J.T., Pope A.L., Ganther H.E., Swanson A.B., Hafeman D.G., Hoekstra W.G. (1973). Selenium: Biochemical role as a component of glutathione peroxidase. Science.

[121] Sun L.L., Gao S.T., Wang K., Xu J.C., Sanz-Fernandez M.V., Baumgard L.H., Bu D.P. (2019). Effects of source on bioavailability of selenium, antioxidant status, and performance in lactating dairy cows during oxidative stress-inducing conditions. J. Dairy Sci..

[122] Sun P., Wang J., Liu W., Bu D.P., Liu S.J., Zhang K.Z. (2017). Hydroxy-selenomethionine: A novel organic selenium source that improves antioxidant status and selenium concentrations in milk and plasma of mid-lactation dairy cows. J. Dairy Sci..

[123] Alhussien M.N., Tiwari S., Panda B.S.K., Pandey Y., Lathwal S.S., Dang A.K. (2021). Supplementation of antioxidant micronutrients reduces stress and improves immune function/response in periparturient dairy cows and their calves. J. Trace. Elem. Med. Biol..

[124] Zhou Y., Jin Y., Yu H., Shan A., Shen J., Zhou C., Zhao Y., Fang H., Wang X., Wang J. (2019). Resveratrol inhibits aflatoxin B1-induced oxidative stress and apoptosis in bovine mammary epithelial cells and is involved the Nrf2 signaling pathway. Toxicon.

[125] Jin L., Yan S., Shi B., Bao H., Gong J., Guo X., Li J. (2014). Effects of vitamin A on the milk performance, antioxidant functions and immune functions of dairy cows. Anim. Feed. Sci. Technol..

[126] Cos P., De Bruyne T., Apers S., Vanden Berghe D., Pieters L., Vlietinck A.J. (2003). Phytoestrogens: Recent developments. Planta Med..

[127] Liu D.Y., He S.J., Liu S.Q., Tang Y.G., Jin E.H., Chen H.L., Li S.H., Zhong L.T. (2014). Daidzein enhances immune function in late lactation cows under heat stress. Anim. Sci. J..

[128] Kansanen E., Jyrkkänen H.K., Levonen A.L. (2012). Activation of stress signaling pathways by electrophilic oxidized and nitrated lipids. Free Radic. Biol Med..

[129] Li L., Yang N., Nin L., Zhao Z., Chen L., Yu J., Jiang Z., Zhong Z., Zeng D., Qi H. (2015). Chinese herbal medicine formula tao hong si wu decoction protects against cerebral ischemia-reperfusion injury via PI3K/Akt and the Nrf2 signaling pathway. J Nat. Med..

[130] Ma Y., Feng Y., Song L., Li M., Dai H., Bao H., Zhang G., Zhao L., Zhang C., Yi J. (2021). Green tea polyphenols supplementation alters immunometabolism and oxidative stress in dairy cows with hyperketonemia. Anim. Nutr..

[131] Khan I., Qureshi M.S., Akhtar S., Ali I., Ghufranullah (2016). Fertility Improvement in Cross-Bred Dairy Cows Through Supplementation of Vitamin E as Antioxidant. Pak. J. Zool..

[132] Wei J.Y., Ma F.T., Hao L.Y., Shan Q., Sun P. (2019). Effect of differing amounts of zinc oxide supplementation on the antioxidant status and zinc metabolism in newborn dairy calves. Livest. Sci..

[133] Ma M., Ma X., Cui J., Guo Y., Tang X., Chen C., Zhu Y., Cui C., Wang G. (2019). Cyclosporin A Protected Cardiomyocytes Against Oxidative Stress Injury by Inhibition of NF-κB Signaling Pathway. Cardiovasc. Eng Technol..

[134] Kang Y., Sun Y., Li T., Ren Z. (2020). Garcinol protects against cerebral ischemia-reperfusion injury in vivo and in vitro by inhibiting inflammation and oxidative stress. Mol. Cell. Probes..

[135] Reuter S., Gupta S.C., Chaturvedi M.M., Aggarwal B.B. (2010). Oxidative stress, inflammation, and cancer: How are they linked?. Free Radic. Biol. Med..

[136] Henley D.V., Bellone C.J., Williams D.A., Ruh M.F. (2004). MAPK signaling pathways modulate IL-1beta expression in human keratinocytes. Arch. Biochem. Biophys..

[137] Gangwani M.R., Kumar A. (2015). Multiple Protein Kinases via Activation of Transcription Factors NF-κB, AP-1 and C/EBP-δ Regulate the IL-6/IL-8 Production by HIV-1 Vpr in Astrocytes. PLoS ONE.

[138] Al-Gayyar M.M., Abdelsaid M.A., Matragoon S., Pillai B.A., El-Remessy A.B. (2011). Thioredoxin interacting protein is a novel mediator of retinal inflammation and neurotoxicity. Br. J. Pharm..

